# Patterns of Outpatient Phecodes Predating the Diagnosis of Systemic Lupus Erythematosus in Taiwanese Women

**DOI:** 10.3390/jcm11185406

**Published:** 2022-09-14

**Authors:** Ming-Chi Lu, Chia-Wen Hsu, Malcolm Koo

**Affiliations:** 1 Division of Allergy, Immunology and Rheumatology, Dalin Tzu Chi Hospital, Buddhist Tzu Chi Medical Foundation, Dalin 622401, Chiayi, Taiwan; 2 School of Medicine, Tzu Chi University, Hualien City 97004, Hualien, Taiwan; 3 Department of Medical Research, Dalin Tzu Chi Hospital, Buddhist Tzu Chi Medical Foundation, Dalin 622401, Chiayi, Taiwan; 4 Graduate Institute of Long-Term Care, Tzu Chi University of Science and Technology, Hualien City 970302, Hualien, Taiwan; 5 Dalla Lana School of Public Health, University of Toronto, Toronto, ON M5T 3M7, Canada

**Keywords:** systemic lupus erythematosus, National Health Insurance Research Database, disease phenotypes, Phecodes, lasso regression

## Abstract

Shortening the time to diagnosis and initiating early treatment are imperative to improve outcomes in patients with systemic lupus erythematosus (SLE). The aim of this case-control study, based on the data from the Taiwan’s National Health Insurance Research Database (NHIRD), was to investigate the patterns of diagnoses of disease phenotypes in female patients with SLE up to eight years prior to its definitive diagnosis. The 547 cases were selected from the 2000–2012 NHIRD catastrophic illness datafile and frequency-matched with 2188 controls. The primary diagnosis based on the first ICD-9-CM code for each outpatient visit was converted to Phecodes. Separate regression models, based on least absolute shrinkage and selection operator (lasso) regularization, with seven different lag periods from 1–2 to 7–8 years, were conducted. Results showed that SLE was associated with 46 disease phenotypes in a lag period of 2–3 years, but fewer in other lag periods. A number of SLE-associated disease phenotypes, such as primary thrombocytopenia, thyroid diseases, Raynaud’s syndrome, renal disease, and several infectious diseases, occurred mainly in the first few years prior to SLE diagnosis. In conclusion, SLE should be suspected when the disease phenotypes identified in the present study occurred concomitantly.

## 1. Introduction

Systemic lupus erythematosus (SLE) is a chronic, heterogeneous, systemic autoimmune disease with multi system involvement. Women, particularly of childbearing age, are more susceptible to the disease [1]. It is currently believed that the onset of SLE is triggered by exposure to certain environmental factors, such as ultraviolet light, infections, and hormonal factors in genetically susceptible individuals [2]. Aberrant epigenetic regulation has been implicated in the pathogenesis of SLE, but the exact mechanism remains unclear [3].

A nationwide population-based study based on the National Health Insurance Research Database showed that the overall prevalence of SLE in Taiwan in 2011 was 8.11 per 10,000 people, with 14.3 per 10,000 women and 1.62 per 10,000 men. The highest prevalence rate was observed at the 40–49 age group in women [4]. SLE is also associated with a substantial economic burden, including the use of health care resource and losses of productivity due to impairment of work capacity [5,6].

The clinical course of SLE is highly variable with recurrent exacerbations of varying severity [7]. Despite recent improvements in the treatment of SLE, disease-related and treatment-related comorbidities can still have a serious and persistent impact on health-related quality of life in patients with SLE [8]. In addition, a multisite international SLE cohort consisting of 9547 patients indicated that while the mortality due to infections and renal disorders had decreased over time, deaths due to circulatory diseases did not change [9].

Currently, diagnosis of SLE is primarily based on clinical and laboratory findings after exclusion of other diseases. Although classification criteria, such as the American College of Rheumatology (ACR) 1997, the Systemic Lupus International Collaborating Clinics criteria (SLICC) 2012, and the European League Against Rheumatism (EULAR)/ACR 2019, designed for research purposes are available, the fulfilment of these classification criteria is not sufficient for a diagnosis of SLE [10].

Initial clinical manifestations of SLE can vary widely. They may involve one or more organ systems and over a variable period. Musculoskeletal, cutaneous, renal, hematological, cardiac, pulmonary, gastrointestinal, ocular, neuropsychiatric, immunologic systems may be involved. Other non-specific symptoms, such as fatigue, fever, arthritis, and arthralgia are commonly present in patients with SLE [11]. Therefore, the diagnosis of SLE has always been considered a clinical challenge for internists [12].

As early clinical features of SLE are mild and non-specific, the interval between the initial symptoms and the diagnosis of SLE can be long. Ozbek et al. reported a mean delay from the first symptom to diagnosis of 21.8 months. The study also revealed that the interval between onset of symptoms and the diagnosis of SLE could depend on the types of initial symptoms. While malar rash (mean delay 6.6 months) or renal involvement (mean delay 15.1.6 months) could lead to early diagnosis of SLE, arthritis and arthralgia (mean delay 23.8 months) was associated with a delay in diagnosis when compared with patients without these symptoms [13]. Kernder et al. found that the mean time to diagnosis was 47 months (median 13 months), including 13 months from the first symptoms to the first physician visit and 34 months from the first physician visit to the diagnosis of SLE [14]. An observational study of 275 Greek patients with SLE found that the median time between the onset of the symptoms and the diagnosis of SLE was 24 months, with patients consulting a mean of three different physicians before achieving the diagnosis [15]. A survey conducted on 2527 patients with SLE in the United Kingdom found a mean diagnosis time from the first symptom of 6.4 years, and 47% of them received a different diagnosis prior to SLE [16].

A cross-sectional analysis of the data from the LuLa study, which is a nationwide survey among Germany’s patients with SLE, revealed that delays in diagnosis were significantly associated with a lower health-related quality of life and greater disease-related damage [14]. Another retrospective longitudinal matched cohort study based on the Thomson Reuters MarketScan database reported that patients diagnosed with SLE sooner could experience lower flare rates, hospitalization rates, and healthcare costs [17]. It is clear that shortening the time to diagnosis and initiating early treatment are imperative to improve outcomes, including preventing irreversible organ damage, in patients with SLE.

In the United Kingdom Clinical Practice Research Datalink study with 1739 incident SLE cases and 6956 controls, Rees et al. reported that patients with SLE consulted their general practitioner more frequently and with clinical features attributable to SLE in the five years preceding diagnosis of SLE [18]. Our previous study, based on data from the Taiwan National Health Insurance Research Database (NHIRD), revealed that patients with SLE had a significantly increased use of medical care in the eight years preceding their diagnosis of SLE. The frequencies of medical visits related to almost all organ systems were significantly higher in patients with SLE compared with controls in the 0.5- to 2-year period preceding the diagnosis of SLE [19]. However, the study did not examine which diagnoses were higher and in which lag period in patients with SLE. Therefore, the aim of this case-control study was to investigate the patterns of diagnosis in female patients with SLE up to eight years before a definitive diagnosis of SLE. Due to the large number of different diagnostic codes, based on the International Classification of Diseases, ninth revision, clinical modification (ICD-9-CM), in relation to the sample size, the least absolute shrinkage and selection operator (lasso) regularization [20] was used to identify influential diagnostic codes. In addition, to better align with the diseases encountered in clinical practice, ICD-9-CM codes were converted to Phecodes in the present analysis [21]. Phecodes was developed for phenome-wide association studies (PheWAS) to deal with the issue that multiple patient billing codes are sometimes used to describe the same clinical disease [22].

## 2. Materials and Methods

### 2.1. Study Design and Data Source

This study used a case-control design based on health claim data from the Taiwan NHIRD, which is a nationwide, population-based database that contains comprehensive medical service utilization records of over 99% of the Taiwanese population [23].

The study protocol was approved by the institutional review board of Dalin Tzu Chi Hospital, Buddhist Tzu Chi Medical Foundation, Taiwan (No. B10104020). The requirement for obtaining informed consent from the patients was waived by the institutional review board because the database contains deidentified information. 

The 2000–2012 catastrophic illness datafile, which is a subset of the NHIRD, was used as the source of patients with SLE. Patients were defined as having SLE if they received a diagnostic code 710.0 based on the International Classification of Diseases, ninth revision, clinical modification (ICD-9-CM) and were also holders of a catastrophic illness certificate. In Taiwan, patients with SLE can apply for a catastrophic illness certificate from the National Health Insurance Administration. The certificate is issued to patients after their medical and serological reports have been reviewed by the National Health Insurance Administration and confirmed to fulfill the 1997 American College of Rheumatology revised criteria for the classification of SLE [24]. Holders of the certificate are exempted from their SLE-related health care copayment fee. 

Female patients with SLE were selected between 1 January 2000 and 31 December 2012. The index date was defined as the date of application of the catastrophic illness certificate. Patients under 20 or over 60 years of age at the index date were excluded from the study.

Controls were randomly sampled from the outpatient datafile of the 2000 Longitudinal Health Insurance Database (LHID 2000) with claim records between 1 January 2000 and 31 December 2012. The LHID 2000 is a subfile of the NHIRD containing health claim data for one million beneficiaries randomly sampled from all enrollees of the NHIRD in 2000. For each patient with SLE, four patients were selected, based on frequency matching for five-year age interval, insured amount, urbanization level, and year of index date.

Payroll-related insured amount was used as a proxy measure of a patient’s socioeconomic status. The variable was categorized into three levels with the lower and upper cut-points set at New Taiwan $19,000 and $24,000, respectively. The urbanization level of a patient’s residence was derived according to a published scheme, which is based on a combination of population density, proportion of residents with college level or higher education, proportion of residents >65 years, proportion of residents who were agriculture workers, and the number of physicians per 100,000 people [25].

### 2.2. Identification of Main Diagnosis of Outpatient Medical Visits 

To investigate the occurrence of various clinical diagnoses before the onset of SLE, the primary diagnosis, represented by the first ICD-9-CM code, for every outpatient visit was retrieved. Since the data used in the present study were between 2000 and 2012, ICD-9-CM instead of ICD-10-CM codes were used. In Taiwan, ICD-9-CM was replaced by ICD-10-CM in 2016 onward. 

The ICD-9-CM codes were converted to Phecodes using the mapping provided on the PheWAS website (https://phewascatalog.org/phecodes, accessed on 23 August 2022). The Phecode system was originally developed to facilitate phenome-wide association studies (PheWAS) by combining one or more related ICD-9-CM codes into distinct and meaningful diseases or traits based on the consensus of clinical experts. Phecodes are arranged hierarchically, similar to the ICD-9-CM codes. However, its hierarchical structure is not based on the billing hierarchy used by ICD-9-CM [26].

A separate variable was created for every Phecode noted in the outpatient visit. Subsequently, these newly created variables were subsequently entered into the regression model to identify influential variables associated with SLE. Seven separate regression models with different lag periods were created according to the date of the outpatient visit. The five lag periods were (1) 1 to 2 years, (2) 2 to 3 years, (3) 3 to 4 years, (4) 4 to 5 years, (5) 5 to 6 years, (6) 6 to 7 years, (7) 7 to 8 years before the index date. All Phecode variables were treated as continuous with their values equal to the frequencies of the outpatient visit for the respective Phecode. In addition, those Phecodes that appeared only once for each patient within each lag period were excluded from the analysis. 

### 2.3. Statistical Analysis

All statistical analyses were performed using Stata Statistical Software, Release 17 (StataCorp, College Station, TX, USA). Continuous variables were summarized as mean with standard deviation (SD) and median with interquartile range, as appropriate. Categorical variables were presented as frequencies and percentages. The basic characteristics between patients with SLE and controls were compared using the chi-square test and the *t*-test, as appropriate.

All regression models (seven separate logistic regression models with different lag periods) were constructed using lasso regularization as the variable selection method. Lasso is a statistical method developed by Tibshirani in 1996 to perform variable selection and regularization to enhance both the accuracy and interpretability of regression models [18]. Instead of using least square methods to fit a model that contains a subset of the predictors, lasso shrinks or regularizes the sum of the absolute values of the regression coefficients to be less than a fixed value. The fixed value is called the shrinkage parameter (λ). In lasso, the regression coefficients of noninfluential predictors are allowed to shrink to zero, which effectively equals to performing variable selection. When λ = 0, the lasso will simply be equal to the least squares fit, and when λ becomes sufficiently large, the lasso will give the null model in which all the coefficient estimates equal zero. The optimal value of λ was estimated by using ten-fold cross-validation. The λ value was selected for which the cross-validation error is the smallest [27].

## 3. Results

The basic characteristics of the 547 patients with SLE and the 2188 controls are shown in Table 1. As expected, no significant differences were observed between the two groups with respect to age, geographic region, and socioeconomic status because of the frequency matching used.

Table 2 shows the top 10 disease phenotypes within each of the seven lag period that were associated with outpatient medical visits in female patients with SLE. The rows were arranged according to Phecode in ascending order, except for the first two rows representing SLE (Phecode 695.42) and cutaneous lupus erythematosus (Phecode 695.41). The ranking of the Phecodes was based on the magnitude of the regression coefficients from the lasso regression. In total, 30 different Phecodes were identified over the seven lag periods. The numbers of the associated Phecodes were the highest in the year 2–3 at 46 and decreased to only 3 in the year 7–8.

SLE (Phecode 695.42) and cutaneous lupus erythematosus (Phecode 695.41) were consistently ranked the top two most influential disease phenotypes associated with SLE in all lag periods. Primary thrombocytopenia (Phecode 287.31) appeared in the first five most recent lag periods. Renal-related disorders, including chronic renal failure (Phencode 585.3), chronic glomerulonephritis, not otherwise specified (Phecode 580.14), nephrotic syndrome without mention of glomerulonephritis (Phecode 580.2), and calculus of ureter (Phecode 594.3) also appeared in the first four most recent lag periods. Other known manifestations of SLE, such as Raynaud’s syndrome (Phecode 443.1), arthropathy not otherwise specified (Phecode 716.9), Graves’ disease (Phecode 242.1), chronic lymphocytic thyroiditis (Phecode 245.21), visual disturbances (Phecode 368), infectious conjunctivitis (Phecode 369.5), dental caries (Phecode 521.1), iron deficiency anemias (Phecode 280.1), and herpes zoster (Phecode 053) appeared only once or twice during different lag periods. Localized superficial swelling, mass, or lump (Phecode 687.2), unspecified diffuse connective tissue disease (Phecode 709.7), and disorders involving the immune mechanism (Phecode 279) appeared only in the lag period of 1–2 years. Sicca syndrome (Phecode 709.2) and systemic sclerosis (Phecode 709.3) appeared only in the lag period of 4–5 years. In addition, musculoskeletal manifestations and bone involvement, including displacement of intervertebral discs (Phecode 722.1), fracture of unspecified bones (Phecode 809), and internal derangement of the knee (Phecode 835) appeared only in the lag period of 5–8 years. Gynecological diseases, including other benign neoplasm of the uterus (218.2) appeared only in the lag period of 5–6 years, whereas inflammatory diseases of the uterus, except cervix (Phecode 614.4) and miscarriage or stillbirth (Phecode 634), appeared only in the lag period of 5–6 years. An unexpected observation is the inverse association between SLE and acute respiratory tract infections, including acute upper respiratory infections of multiple or unspecified sites (Phecode 465) and acute bronchitis and bronchiolitis (Phecode 483).

**Figure 1:**
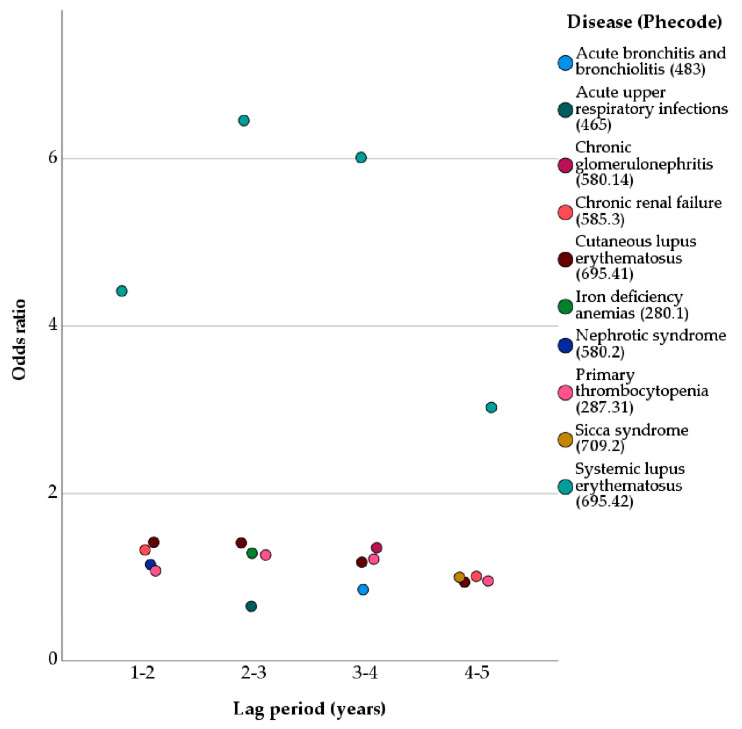
Top five disease phenotypes with the first four most recent lag periods that were associated with outpatient medical visits in women with systemic lupus erythematosus.

The data shown in Table 2 were arranged visually to show the top five disease phenotypes within each of the first four most recent lag periods that were associated with outpatient medical visits in female patients with SLE (Figure 1). The y-axis showed the odds ratio for each of the disease phenotypes at the four different lag periods. All the odds ratios for the associations were below 2 except for systemic lupus erythematosus (695.42).

## 4. Discussion

Our previous study, based on the data from the Taiwan NHIRD, showed that patients with SLE had diseases of the respiratory system, digestive system, musculoskeletal system, and skin tissue several years preceding the definitive diagnosis of SLE [19]. The present study further explored the specific disease phenotypes associated with SLE between one and seven years before the definitive diagnosis of SLE. In this study, we excluded the analysis of outpatient visit records within one year before the definitive diagnosis of SLE because most patients with SLE would receive relevant laboratory tests within a one-year period prior to the diagnosis of SLE [19]. As ICD-9-CM was developed with a hierarchical structure for billing purposes, the present study used Phecodes instead, in an attempt to achieve more meaningful representation of diseases or traits observed in clinical settings. In a study comparing 100 disease phenotypes based on known associations for 440 single-nucleotide polymorphism (SNP)-phenotype pairs, it was found that Phecodes could provide groupings of disease codes that are better aligned with clinical diseases mentioned in clinical practice than ICD-9-CM codes [26]. Another unique aspect of this study was the use of lasso regularization instead of *p*-value-based variable selection in a sparse logistic model to avoid the issue of overfitting and to deal with high multicollinearity among the independent variables.

Overall, different disease phenotypes appeared within a lag period up to five years might be considered as a diagnostic time window to prompt clinicians to suspect SLE. Those appeared earlier were too few in number and non-specific to be informative. The number of influential disease phenotypes was generally higher at a time closer to the date of definitive diagnosis of SLE. At a lag period of 1–2 years, SLE was associated with 22 disease phenotypes, and the number increased to 46 and 40 with 2–3 and 3–4, respectively. However, the number reduced to less than 10 with a lag time of five years or more. At a lag period of 6–7 years, in addition to disease phenotype SLE (Phecode 695.42) and cutaneous lupus erythematosus, SLE was only associated with fracture of unspecified bones and primary thrombocytopenia. The latter finding is consistent with a population-based retrospective cohort study, which reported a significant increased risk of SLE in patients with idiopathic thrombocytopenic purpura [28]. While the increased risk of incident fracture and osteoporosis is observed in patients with SLE [29], the reasons for the association between SLE and fracture of unspecified bones at a lag period of 6–7 years as well as displacement of intervertebral discs at a lag period of 7–8 years are unclear and will require further elucidation.

The disease phenotypes SLE and cutaneous lupus erythematosus were consistently and strongly associated with SLE in all seven lag periods. It is expected that patients would eventually be suspected of having SLE and confirmed with follow-up laboratory tests when the time is close to the definitive diagnosis. However, it is of interest to note that even 7 to 8 years prior to its definitive diagnosis, SLE was already noted as a primary diagnosis in these patients. Additional studies will be required to explore whether the long duration before the final recognition of SLE in these patients was the result of milder presentations in the early stage of the disease, and therefore, these patients were not referred to a rheumatologist for follow-up.

SLE was also associated with primary thrombocytopenia in five lag periods (1–2, 2–3, 3–4, 4–5, and 6–7 years). Thrombocytopenia is one of the hematological criteria of SLE, which is defined as a platelet count of <100,000/mm^3^, according to the ACR classification criteria [30]. It is known that patients with thrombocytopenia are more prone to exhibit renal [31] and hematological manifestations [32]. In the present study, SLE was associated with iron deficiency anemia, chronic renal failure, chronic glomerulonephritis, nephrotic syndrome, and calculus of ureter, and the timing was indeed coincided with that of primary thrombocytopenia in the first four more recent lag periods. The diagnosis of calculus of ureter could be explained by the presence of hematuria as a result of nephritis.

Among other common manifestations of SLE, most of them appeared in the three lag periods between 2 and 5 years, including herpes zoster [33], infectious conjunctivitis [34], Raynaud’s syndrome [35], dental caries [36], and local infections of skin and subcutaneous tissue [37]. Moreover, overlapping of SLE and other systemic autoimmune diseases could be observed with a lag period of 4–5 years. SLE was associated with sicca syndrome and systemic sclerosis. SLE is known to coexist with Sjögren’s syndrome with a prevalence of 14% [38] and with systemic sclerosis with a prevalence of 6.8% [39]. The overlapping clinical manifestations and similar autoantibody profile between Sjögren’s syndrome and SLE have been suggested as a result of shared underlying etiopathogenic aspects, including genetic factors, epigenetic, environmental, and hormonal factors between the two disorders [40,41]. It is plausible that the presence of Sjögren’s syndrome prior to SLE could play a role in the association of SLE with dental caries and infectious conjunctivitis. The significant associations between SLE with dental caries and infectious conjunctivitis were previously shown in studies based on the Taiwan NHIRD. A case-control study revealed that the utilization of eye disorder outpatient medical services and dental services was significantly higher in patients with Sjögren’s syndrome several years before the diagnosis of the disease [42,43]. It has been shown that abnormal regulation of the T helper (Th) 17 cell pathway could be related to the occurrence of dry eye disease in patients with SLE and Sjögren’s syndrome [44]. A study based on the Taiwan NHIRD showed that dry eye syndrome could occur before the diagnosis of Sjögren’s syndrome with a median time of approximately four years [45]. While about 10% of patients with clinically significant aqueous deficient dry eye has underlying Sjögren’s syndrome, the disease is often underdiagnosed [46]. Ophthalmologists should be vigilant about the association between dry eye symptom and autoimmune rheumatic diseases to prevent delays in diagnosis.

Thyroid-related diseases, including chronic lymphocytic thyroiditis, occurred with a lag period of 2–3 and 4–5 years, and Graves’ disease occurred with a lag period of 5–6 years. A secondary retrospective study based on the Taiwan NHIRD reported that hyperthyroidism, hypothyroidism, and autoimmune thyroid disease were significantly higher in patients with SLE [47]. Graves’ disease was found to be significantly associated with an increased risk of incident SLE in another study based on the Taiwan NHIRD. The authors suggested that the SLE and Graves’ disease might have a shared genetic predisposition, and it is also possible that antithyroid medications used in the treatment of Graves’ disease could induce SLE [48].

SLE was associated with arthropathy and internal derangement of the knee in the lag period of 5–6 years. These conditions were in line with joint involvement commonly seen in patients with SLE [49]. SLE was also associated with three gynecological disorders, including miscarriage/stillbirth and inflammatory diseases of the uterus, except the cervix, in the lag period of 5–6 years and other benign neoplasm of the uterus in the lag period of 4–5 years. While the reasons for the latter two associations will require further investigation, the association with miscarriage and stillbirth could be explained by the coexistence of anti-phospholipid syndrome (APS) and SLE. APS is an autoimmune disease characterized by vascular thrombosis or obstetric complications, such as miscarriage and stillbirths [50]. Previous research suggested that APS could also be a forerunner of SLE [51]. A recent cohort study based on the Taiwan NHIRD reported that the risk for incident SLE in the APS group was 28 times higher than the non-APS group after propensity score-matching [52].

In this study, SLE was associated with fewer outpatient visits for acute respiratory tract infections. This is an unexpected finding, because the impaired immune functions and the use of immunosuppressive agents in SLE should contribute to the vulnerability to infection [53]. A prospective study on 110 outpatients with SLE reported that these patients were especially prone to develop urinary infection, pneumonia, and bacteremia without focus [54]. Another case-control study of 83 patients with SLE showed that pneumonia and bacteremia occurred in 41% and 24% of the patients, respectively [55]. However, these findings might not be directly compared with those in the present study because the observation periods were different. Whether a lower frequency of outpatient visits for acute respiratory tract infections prior to SLE diagnosis is due to a different healthcare- seeking behavior or an increasing number of different diseases competing for primary diagnosis will require further investigation.

Furthermore, our studies did not find that manifestations common in SLE occurred prior to its definitive diagnosis. For example, disease phenotypes related to heart involvement [56] and neurologic involvement [57] were not observed in any of the lag periods. This is consistent with the finding of our previous study that the four most common organ system involvement of SLE did not include the circulatory system and the nervous system [19].

Our study has some limitations that deserve to be mentioned. First, the definitive diagnosis of SLE was defined as the application date of the catastrophic illness certificate for SLE. Our previous study estimated that there was a mean lag time of 12 days between the index date and the definitive diagnosis of SLE, which was defined as three consecutive visits with the diagnosis of SLE. However, we have excluded the medical records within the first year prior to the index date and, therefore, the short lag time of 12 days should not materially affect our conclusion. Second, information on SLE disease severity was not available, which is a limitation inherent in the use of NHIRD for analysis.

Despite the limitations, this study had several strengths. To our knowledge, this study was the first to explore disease phenotypes associated with SLE in various lag periods before the definitive diagnosis of SLE. Second, a multicenter cross-sectional study of 300 patients with SLE reported that 30.3% of the patients were seen by two physicians and 18% were seen by five or more physicians before the diagnosis of SLE was made [58]. Therefore, the use of the NHIRD, which is a nationwide, population-based database, is necessary to obtain a complete healthcare seeking records of patients with SLE over time because their medical consultations might span across different clinics and hospitals. Third, logistic regression with lasso regularization was used to overcome the difficulties in modeling the data with sparse structure.

In conclusion, SLE is a complex autoimmune disease with variable manifestations. Our findings suggested that a period of up to five years before the definitive diagnosis of SLE might be an informative diagnostic time window. An increasing number of different diseases occurred during this period. While it is known that many of the SLE symptoms, such as fatigue, rash, and fever, are non-specific and overlap with other common disorders, the present study revealed a number of specific disease phenotypes that clustered within a relatively short period of time. SLE should be suspected when these disease phenotypes occurred concomitantly. These patients should be referred to a rheumatologist for confirmation to aid early diagnosis and treatment of SLE.

## Figures and Tables

**Table 1:** Basic characteristics of patients with systemic lupus erythematosus and controls (*n* = 2735).

Variable	*n* (%)		*p* Value
	Systemic lupus erythematosus 547 (20.0)	Control 2188 (80.0)	
Age group, years			>0.999
20–29	170 (31.1)	680 (31.1)	
30–39	161 (29.4)	644 (29.4)	
40–49	127 (23.2)	508 (23.2)	
50–59	89 (16.3)	356 (16.3)	
Mean age, years (SD)	36.8 (10.6)	36.8 (10.6)	>0.999
Socioeconomic status			>0.999
Low	182 (33.3)	728 (33.3)	
Medium	136 (24.9)	544 (24.9)	
High	229 (41.9)	916 (41.9)	
Urbanization level			>0.999
Urban	140 (25.6)	560 (25.6)	
Suburban	135 (24.7)	540 (24.7)	
Rural	272 (49.7)	1088 (49.7)	

Socioeconomic status was estimated by insurance premiums based on salary. Low: <19,000 New Taiwan dollars (NT$); middle: 19,001–24,000; and high: >24,000. SD: standard deviation.

**Table 2:** Top 10 disease phenotypes within each lag period that were associated with outpatient medical visits in women with systemic lupus erythematosus.

Disease Phenotype (Phecode)	Years Prior to Definitive Diagnosis of SLE						
	1–2	2–3	3–4	4–5	5–6	6–7	7–8
Systemic lupus erythematosus (695.42)	1	1	1	1	1	1	1
Cutaneous lupus erythematosus (695.41)	2	2	2	2	2	2	2
Herpes zoster (053)		6					
Other benign neoplasm of uterus (218.2)				7			
Graves’ disease (242.1)					3		
Chronic lymphocytic thyroiditis (245.21)		10		10			
Disorders involving the immune mechanism (279)	6						
Iron deficiency anemias, unspecified or not due to blood loss (280.1)		4	6				
Thrombocytopenia (287.3)		7					
Primary thrombocytopenia (287.31)	5	5	4	3		4	
Visual disturbances (368)	10						
Conjunctivitis, infectious (369.5)			10				
Raynaud’s syndrome (443.1)			7	8			
Acute upper respiratory infections of multiple or unspecified sites (465)		3 ↓			7 ↓		
Acute bronchitis and bronchiolitis (483)	7 ↓						
Dental caries (521.1)			3				
Chronic glomerulonephritis, not otherwise specified (580.14)		9	5				
Nephrotic syndrome without mention of glomerulonephritis (580.2)	4						
Chronic renal failure (585.3)	3	8		4			
Calculus of ureter (594.3)				9			
Inflammatory diseases of uterus, except cervix (614.4)					5		
Miscarriage; stillbirth (634)					6		
Other local infections of skin and subcutaneous tissue (686)			9				
Localized superficial swelling, mass, or lump (687.2)	8						
Sicca syndrome (709.2)				5			
Systemic sclerosis (709.3)				6			
Unspecified diffuse connective tissue disease (709.7)			8				
Arthropathy, not otherwise specified (716.9)	9				8		
Displacement of intervertebral disc (722.1)							3
Fracture of unspecified bones (809)						3	
Internal derangement of knee (835)					4		
**Number of non-zero coefficient**	**22**	**46**	**40**	**25**	**8**	**4**	**3**

SLE: systemic lupus erythematosus; ↓: inverse association. The rows in the table are arranged according to the Phecodes in ascending order, except for the first two rows (systemic lupus erythematosus and cutaneous lupus erythematosus).

## Data Availability

The data are not publicly available due to the Taiwan Personal Information Protection Act. The data that support the findings of this study are available on request from the corresponding author.
